# Isoform a4 of the vacuolar ATPase a subunit promotes 4T1-12B breast cancer cell–dependent tumor growth and metastasis *in vivo*

**DOI:** 10.1016/j.jbc.2022.102395

**Published:** 2022-08-19

**Authors:** Kevin Su, Michael P. Collins, Christina M. McGuire, Mohammed A. Alshagawi, Mariam K. Alamoudi, Zhen Li, Michael Forgac

**Affiliations:** 1 Department of Pharmacology and Drug Development, Graduate School of Biomedical Sciences, Tufts University, Boston, Massachusetts, USA; 2 Department of Cellular, Molecular and Developmental Biology, Graduate School of Biomedical Sciences, Tufts University, Boston, Massachusetts, USA; 3 Department of Biochemistry, Graduate School of Biomedical Sciences, Tufts University, Boston, Massachusetts, USA; 4 Department of Developmental, Molecular, and Chemical Biology, Tufts University School of Medicine, Boston, Massachusetts, USA

**Keywords:** vacuolar ATPase, breast cancer, *in vivo* imaging, tumor growth, tumor metastasis, a1–a4, V-ATPase subunit a, isoforms a1 through a4, DMEM, Dulbecco’s modified Eagle medium, FBS, fetal bovine serum, V0, V-ATPase membrane domain, V1, V-ATPase peripheral domain, V-ATPase, vacuolar H+-ATPase

## Abstract

The vacuolar H^+^-ATPase (V-ATPase) is an ATP-dependent proton pump that governs the pH of various intracellular compartments and also functions at the plasma membrane in certain cell types, including cancer cells. Membrane targeting of the V-ATPase is controlled by isoforms of subunit a, and we have previously shown that isoforms a3 and a4 are important for the migration and invasion of several breast cancer cell lines *in vitro*. Using CRISPR-mediated genome editing to selectively disrupt each of the four a subunit isoforms, we also recently showed that a4 is critical to plasma membrane V-ATPase localization, as well as *in vitro* migration and invasion of 4T1-12B murine breast cancer cells. We now report that a4 is important for the growth of 4T1-12B tumors *in vivo*. We found that BALB/c mice bearing a4^−/−^ 4T1-12B allografts had significantly smaller tumors than mice in the control group. In addition, we determined that a4^−/−^ allografts showed dramatically reduced metastases to the lung and reduced luminescence intensity of metastases to bone relative to the control group. Taken together, these results suggest that the a4 isoform of the V-ATPase represents a novel potential therapeutic target to limit breast cancer growth and metastasis.

Breast cancer is the leading cause of cancer-related mortality in women worldwide (1). Significant advancements have been made in the management of localized, early stage breast cancer, with cure rates between 70 and 80% (2). However, late stage, metastatic breast cancer that has spread beyond the lymph nodes is considered to be incurable (2). Metastasis is a multistep process in which cancer cells detach from the primary tumor, degrade ECM, enter the circulatory or lymphatic systems, and extravasate in order to colonize distant sites (3, 4, 5). Several of these steps require an invasive phenotype, which the vacuolar H^+^-ATPase (V-ATPase) has been shown to promote (6, 7). The V-ATPase is a multisubunit rotary machine composed of a peripheral V_1_ domain that hydrolyzes ATP and an integral V_0_ domain that transports protons (8, 9, 10, 11, 12, 13, 14, 15). V-ATPases are present in all eukaryotes where they govern the luminal pH of various intracellular compartments. Certain specialized cells, such as osteoclasts and renal alpha intercalated cells, also localize V-ATPases to the plasma membrane in order to transport protons extracellularly (8, 10, 11, 13).

The role of the V-ATPase in promoting invasion has been most thoroughly characterized in breast cancer, where pharmacological V-ATPase inhibition was found to reduce *in vitro* migration and invasion of numerous breast cancer cell lines (16, 17, 18, 19, 20, 21, 22). Treatment of 4T1 cells with the V-ATPase inhibitor archazolid prior to intravenous injection was also found to reduce lung colonization in mice (22, 23). While these studies were conducted with membrane-permeant inhibitors, thus inhibiting all cellular V-ATPases, we recently showed that specific inhibition of plasma membrane V-ATPases is comparably efficacious *in vitro*. Treatment of highly invasive MDA-MB-231 cells with either a membrane-impermeant form of the V-ATPase inhibitor bafilomycin or with an antibody against an extracellular V5 epitope fused to the V-ATPase c subunit were as effective at inhibiting *in vitro* migration and invasion as pan-V-ATPase inhibition (20).

Membrane targeting of the V-ATPase is controlled by isoforms of subunit a, with a3-containing V-ATPases being targeted to the plasma membrane of osteoclasts, while a4-containing V-ATPases localize to the plasma membrane of kidney α-intercalated cells and epididymal clear cells (24, 25, 26). We previously showed that a3 is critical for the invasiveness of MDA-MB-231 and MCF10CA1a cells, with RNAi-mediated knockdown of a3 or a3+a4 (but not a1 or a2), reducing invasion and plasma membrane V-ATPase localization in these lines (18, 19). Furthermore, transient overexpression of a3 (but not other a isoforms) increased the invasiveness of otherwise noninvasive MCF10a cells (19). These findings appear to be physiologically relevant as a3 (and to a lesser extent, a4) was found to be overexpressed in 43 out of 43 human breast cancer samples, relative to normal mammary epithelial tissue (16).

In order to test the hypothesis that a isoforms are important for *in vivo* tumor cell metastasis, we elected to utilize the 4T1-12B murine metastatic breast cancer line. 4T1 cells were originally derived from a metastatic lesion that arose in the lung of a BALB/c mouse, after serially passaging an MMTV-induced mammary tumor (27, 28, 29). Because of its capacity to metastasize to many of the same sites as human breast cancer, the 4T1 cell line has been widely adopted as a relevant model of late stage breast cancer (30, 31). 4T1-12B cells stably express firefly luciferase, while retaining the metastatic properties of the parental 4T1 line (32). Using CRISPR-mediated genome editing, we previously showed that in 4T1-12B cells, a4 is critical for *in vitro* invasion and plasma membrane localization, while knockout of other isoforms had no effect on these parameters (21).

Here we describe the first use of a isoform knockout cells in an *in vivo* tumor model. We report that knockout of a4, but not any other a isoform, significantly reduced tumor growth *in vivo*, relative to a negative control line. By contrast, knockout of a2 significantly increased tumor growth. Importantly, knockout of a4 nearly eliminated metastasis to the lung while also reducing the size of metastases to bone. These results suggest that a4, which targets the V-ATPase to the cell surface, represents an important potential target in the development of drugs to limit breast cancer growth and metastasis.

## Results

### Luciferase activity is retained in CRISPR-modified 4T1-12B cells

**Figure 1:**
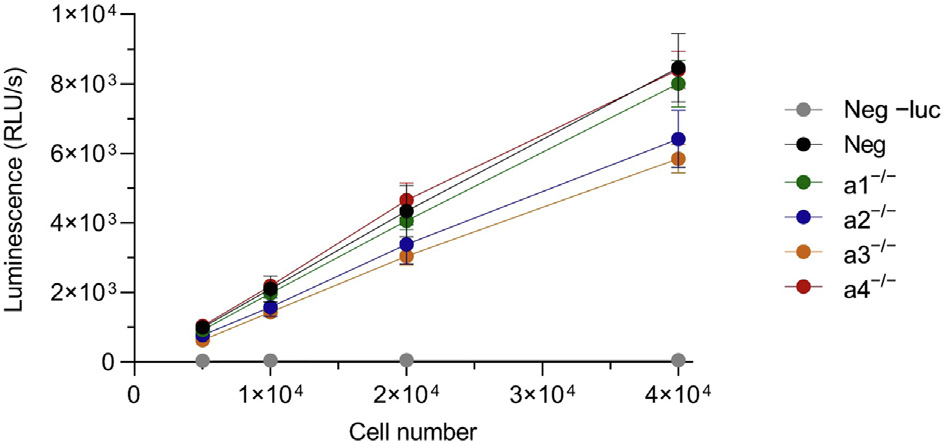
**Luciferase activity of negative control and a isoform knockout 4T1-12B cells *in vitro*.** The indicated cell lines were seeded in triplicate at increasing density. The following day, d-luciferin or vehicle control (−luc) were added, and luminescence was measured as described in Experimental Procedures. Values represent the mean of two independent trials. Error bars represent SEM.

As described previously, we have derived clonal 4T1-12B lines in which each of the genes encoding the four isoforms of the a subunit were selectively disrupted by CRISPR-mediated editing (21). The functional studies described in McGuire *et al.* were performed on the clones showing the most complete loss of the targeted isoform and the least disruption of the other isoforms, as measured by Western blot. These same clones, designated a1^−/−^, a2^−/−^ a3^−/−^ a4^−/−^, as well as a nontargeted CRISPR negative control clone, were used in the present study. Before testing these CRISPR-modified cells in mice, we first wished to confirm that they retain functional luciferase expression. As shown in Fig. 1, luciferase activity was detected in all clones by microplate assay, with no significant differences between any of the groups.

All tumor cells employed were engineered to express luciferase in order to allow the visualization of the tumor cells following implantation in the mammary fat pad and to permit identification of metastases in organs following sacrifice and dissection of mice. Injection of mice with luciferin generated a light signal which was visualized using the IVIS imaging system, as described in the Experimental Procedures section. That the intensity of the signal was proportional to the number of tumor cells present is supported by the agreement in tumor volume measured using either caliper measurement or primary tumor luminescence (Supplemental Fig. 1). Organs were scored as either positive for metastases where the signal was above background or negative for metastases where the signal was the same as background.

### a4^−/−^ 4T1-12B cells form significantly smaller tumors in mice than control cells

Since all clones displayed robust luciferase activity, we proceeded with *in vivo* tumor cell engraftment. Tumors cells were implanted orthotopically in 6-week-old, female BALB/c mice by injecting cell suspensions into the intact no. 4 inguinal fat pad. 10 mice were used per group in each of two separate trials, and tumor progression was monitored for 6 weeks or until humane endpoint criteria were met, as described in Experimental Procedures. Starting at day 7, the primary tumor volumes were determined by external caliper measurement every 3 to 7 days. At day 10 of the first trial, 46 out of 50 mice presented with palpable tumors at the injection site, while one mouse in the negative control group, two mice in the a1^−/−^ group, and one mouse in the a4^−/−^ group did not have palpable tumors. The mean tumor volume for all 50 mice was 91 ± 14 mm^3^, with no statistically significant differences between any of the groups at day 10. Of the four mice that did not have palpable tumors at day 10, only the mouse in the a4^−/−^ group failed to develop a palpable tumor by the next measurement (this mouse remained tumor-free for the duration of the study). Thus, for the first trial, the take rate was 90% (9/10) in the a4^−/−^ group, while all other groups had 100% (10/10) take rates. At day 10 of the second trial, 49 of 49 mice presented with palpable tumors at the injection site. Only nine mice were injected for the a3^−/−^ group because there were not enough cells harvested on study day 0. Thus, the take rate was 100% for the second trial.

**Figure 2:**
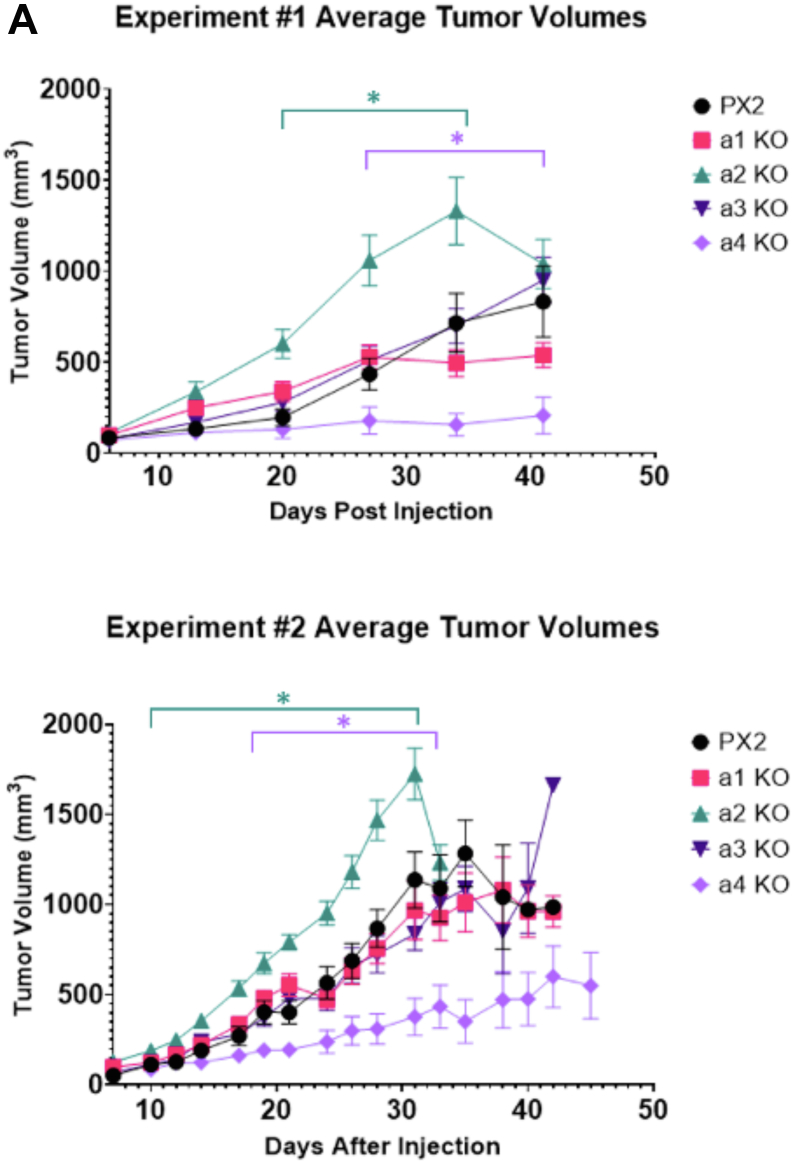

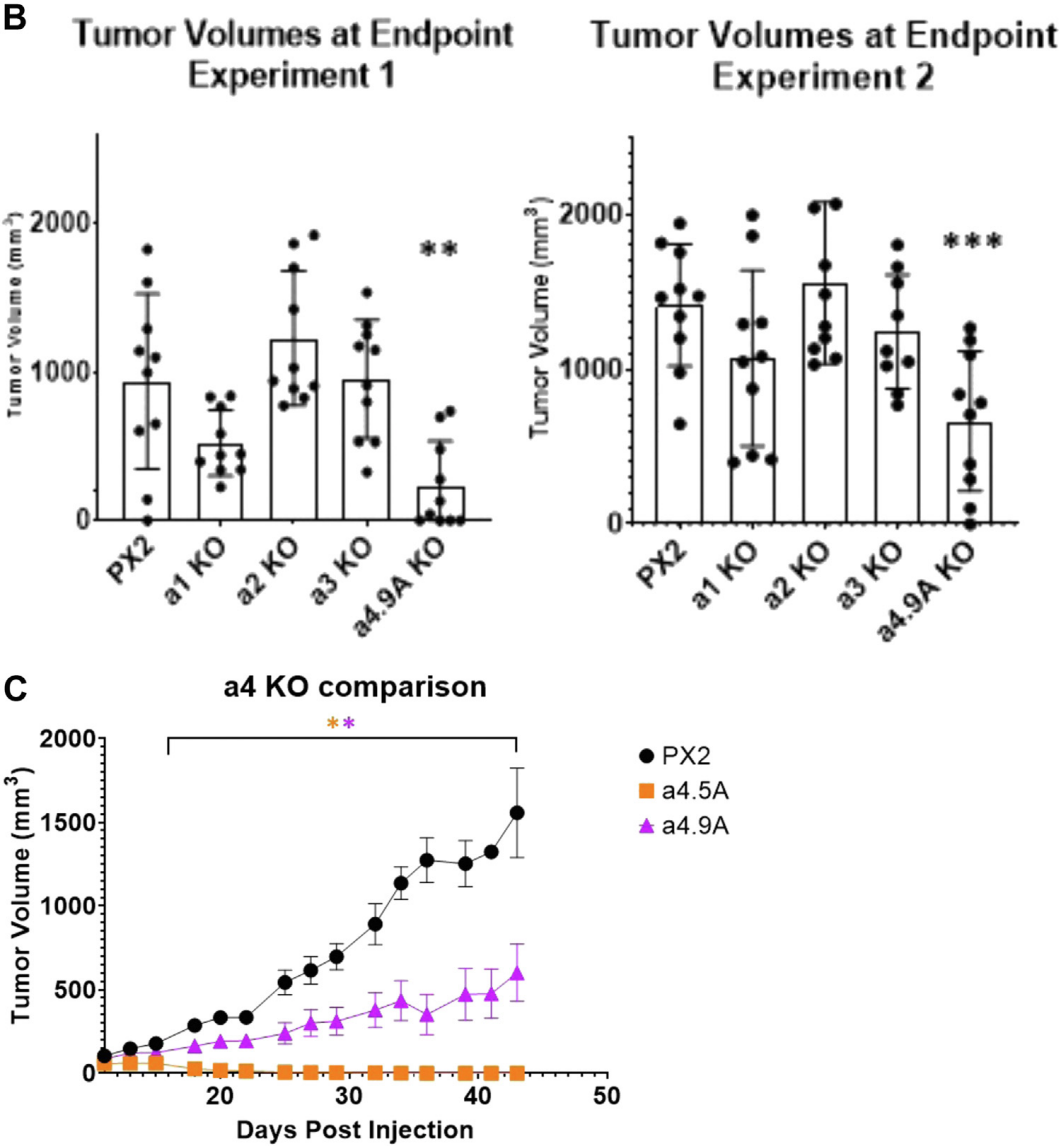
**Primary tumor volumes in BALB/c mice bearing negative control and a isoform knockout 4T1-12B allografts.** CRISPR negative control and a isoform knockout 4T1-12B cells were injected into the intact mammary fat pads of female BALB/c mice, as described in Experimental Procedures. *A*, primary tumor volumes were determined by caliper measurements taken weekly for experiment 1 and three times a week for experiment 2. Values represent the mean. (∗ *p* < 0.05, error bars represent SEM.) *B*, tumor volumes for all mice recorded at time of takedown. (∗∗ *p* < 0.005, ∗∗∗ *p* < 0.0005, error bars represent SEM). *C*, A further trial comparing tumor growth in mice implanted with control (PX2) cells or with the original a4 knockout clone (a 4.9 A) or a second a4 knockout clone (a 4.5 A) was performed (∗ *p* < 0.05, error bars represent SEM). (For experiment 1, n = 10 for PX2, n = 10 for a1 KO, n = 10 for a2 KO, n = 10 for a3 KO, n = 10 for a4 KO; for experiment 2, n = 10 for PX2, n = 10 for a1 KO, n = 10 for a2 KO, n = 9 for a3 KO, n = 10 for a4 KO; For C, n = 5 for PX2, n = 10 for a4.5 A KO, n = 10 for a4.9 A KO).

The growth curves for all mice in each individual trial are shown in Fig. 2*A*, with *asterisks* indicating mean tumor volumes statistically different from the negative control group at a given timepoint (*p* < 0.05). For experiment 1, the a4^−/−^ group had smaller tumors than the control group starting day 27 and persisting through week 6, while a2^−/−^ tumors were larger than controls starting at day 20 and persisting through week 5 (Fig. 2*A*). Results were similar for experiment 2. Again, the a4^−/−^ group had smaller tumors than the control group, with statistical significance being reached at day 17, while the a2^−/−^ group had significantly larger tumors than negative controls starting at day 14 (Fig. 2*A*). Note that throughout the 6 weeks duration of each study, several mice were euthanized due to low body condition score or tumor volumes exceeding 1500 mm^3^. Statistical significance was lost toward the end of both studies as too many mice were taken down. For experiment 1 control group, one mouse was taken down in week 5. For the a1^−/−^ and a3^−/−^ groups, no mice were taken down early. For the a2^−/−^ group, three mice were taken down, and one mouse was found dead in week 4, while another two were taken down in week 5. For the a4^−/−^ group, one mouse was found dead in week 5. For experiment 2 control group, three mice were taken down in week 4 and five mice in week 5. For the a1^−/−^ group, two mice were found dead in week 3, one mouse was taken down in week 4, and one in week 5. For the a2^−/−^ group, one mouse was taken down in week 3, five in week 4, and no mice survived to week 6. For the a3^−/−^ group, eight mice were taken down in week 5. For the a4^−/−^ group, two mice were taken down in week 5.

Tumor volumes for each mouse were recorded at takedown. For experiment 1, the mean tumor volume was 1221 ± 104 mm^3^ in the control group and 439 ± 93 mm^3^ in the a4^−/−^ group, representing a 64% reduction in tumor growth (Fig. 2*B*). To confirm the effect observed of a4 knockout on tumor growth, an additional a4 knockout clone derived by CRISPR/Cas9 disruption of a4 in 4T1-12B cells (a 4.5 A) was implanted and monitored relative to control (PX2) and the original a4 knockout clone (a 4.9 A). As can be seen in Fig. 2*C*, tumors derived from the a 4.5 A knockout clone grew even more slowly than those derived from the a 4.9 A knockout clone. Tumors from both a4 knockout groups were smaller than control tumors by week 3, thus confirming the inhibitory effect of a4 knockout on tumor growth *in vivo*.

### a4^−/−^ 4T1-12B cells showed reduced metastases in mice relative to control cells

As discussed above, an important rationale behind studying the effect of a isoform knockouts in 4T1-12B cells is that the 4T1 model is known to readily metastasize in immunocompetent mice. Thus, in addition to monitoring the growth of the primary tumors, metastasis was measured at the conclusion of the study by sacrificing of mice and removal of the following tissues for *ex vivo* imaging: brain, heart, kidneys, liver, lungs, spleen, and hind legs. Consistent with previous reports, acute splenomegaly and hepatomegaly were observed in all tumor-bearing mice (33, 34). Because of signal interference from the excised primary tumor, metastasis data were not obtained on the initial trial group of 50 mice. For the second trial group, the data are shown in Table 1. As shown, all mice implanted with the PX2 control cells developed metastases in lung and bone and also showed limited metastases in brain, kidney, liver, and spleen. By contrast, the initial a4 knockout clone analyzed (a 4.9 A) showed no metastases to any site other than bone. For the a 4.5 A knockout clone, only one out of nine mice developed metastasis to lung but were otherwise free of metastases outside of bone. Interestingly, mice implanted with the a2 knockout cells developed metastases with approximately the same frequency as mice implanted with the control cells, despite the more rapid growth of the primary tumor. In addition, knockout of a1 also significantly reduced metastasis to the lung (3 out of 8 mice).

**Table 1:** Comparison of frequency of metastases to various organs in BALB/c mice bearing negative control and a isoform knockout 4T1-12B allografts

Cell line	Spleen	Liver	Kidney/adrenal	Lungs	Heart	Brain	Limbs
PX2	3 (15)	3 (15)	2 (15)	15 (15)	0 (15)	1 (15)	15 (15)
a1 KO	0 (8)	1 (8)	0 (8)	3 (8)	0 (8)	0 (8)	8 (8)
a2 KO	1 (10)	2 (10)	1 (10)	10 (10)	0 (10)	0 (10)	10 (10)
a3 KO	0 (9)	0 (9)	0 (9)	9 (9)	0 (9)	0 (9)	9 (9)
a4.5 KO	0 (10)	0 (10)	0 (10)	0 (10)	0 (10)	0 (10)	10 (10)
a4.9 KO	0 (9)	0 (9)	0 (9)	1 (9)	0 (9)	0 (9)	9 (9)

Table shows the frequency of metastases seen in organs imaged *ex vivo* using the IVIS imaging system following implantation of a subunit knockout strains of 4T1-12B cells in mice as described in Experimental Procedures. Data represent the number of mice showing metastases, with the total number of mice per group shown in parentheses.

**Figure 3:**
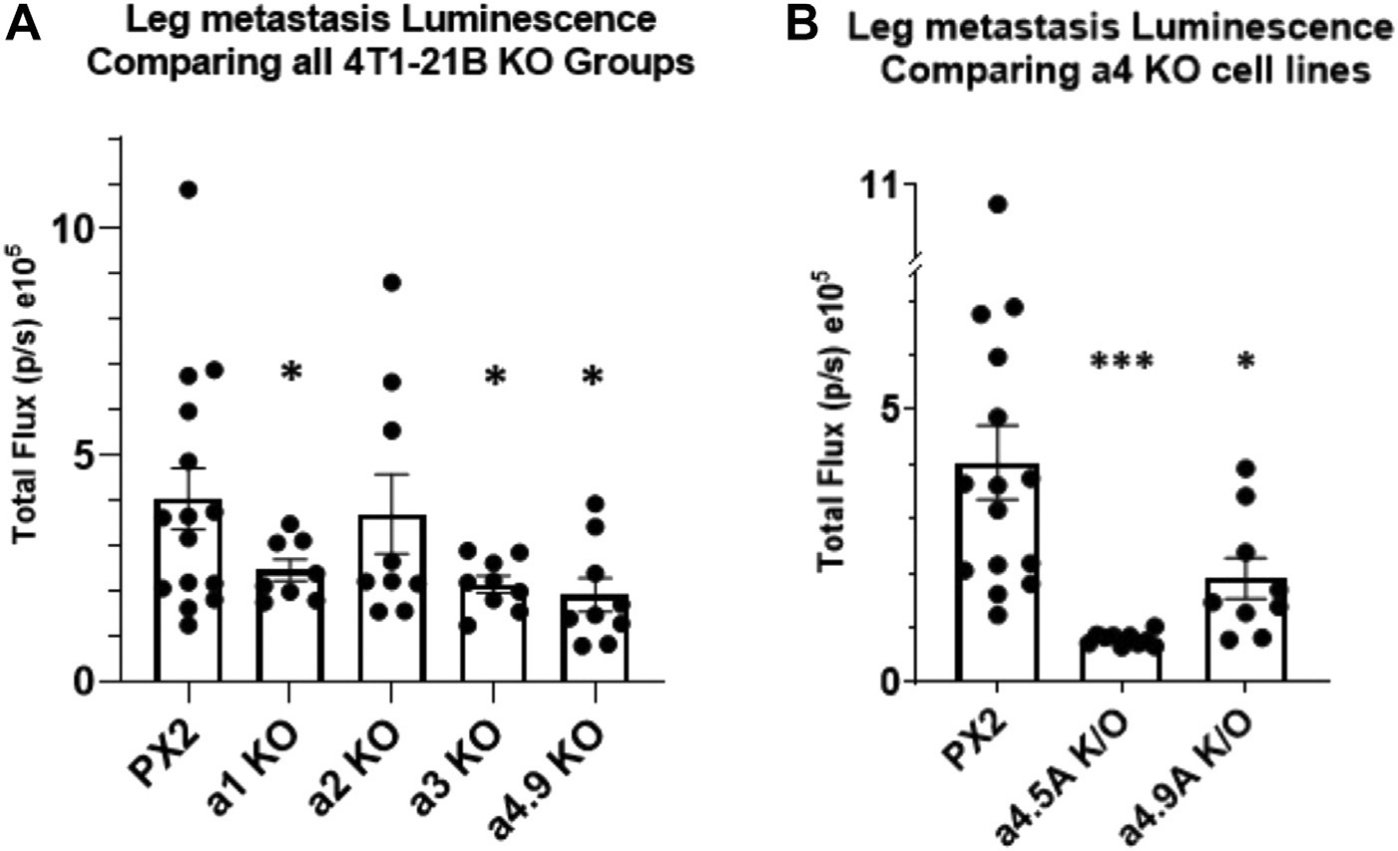
**Luminescence intensity of bone metastases in BALB/c mice bearing negative control and a isoform knockout 4T1-12B allografts .** At study endpoint or after reaching humane endpoint criteria, mice were injected with luciferin as mentioned above. Mice were humanely sacrificed, then hind limbs were imaged. *A*, luminescence measured in hind limbs comparing KO clones of each subunit a isoform. (∗ *p* < 0.05, error bars represent SEM). *B*, luminescence measured in hind limbs comparing control to two different a4 KO clones. (∗ *p* < 0.05, ∗∗∗ *p* < 0.0005, error bars represent SEM) (n = 15 for PX2, n = 8 for a1 KO, n = 10 for a2 KO, n = 9 for a3 KO, n = 10 for a4/a4.9 A KO, n = 9 for a4.5 A KO).

To further evaluate the effect of a isoform knockout on tumor metastasis, the luminescence intensity of bone metastases were compared. As shown in Fig. 3*A*, knockout of all isoforms except a2 reduced the luminescence intensity of bone metastases relative to control cells. Further, as shown in Fig. 3*B*, luminescence intensity of bone metastases derived from the a 4.5 A knockout clone were even further reduced relative to the a 4.9 A knockout clone. These results suggest that although tumor metastasis to bone still occurs in a4 knockout cells, there is a reduced tumor burden in bone with knockout of a4 and, to a lesser extent, a1 and a3.

**Figure 4:**
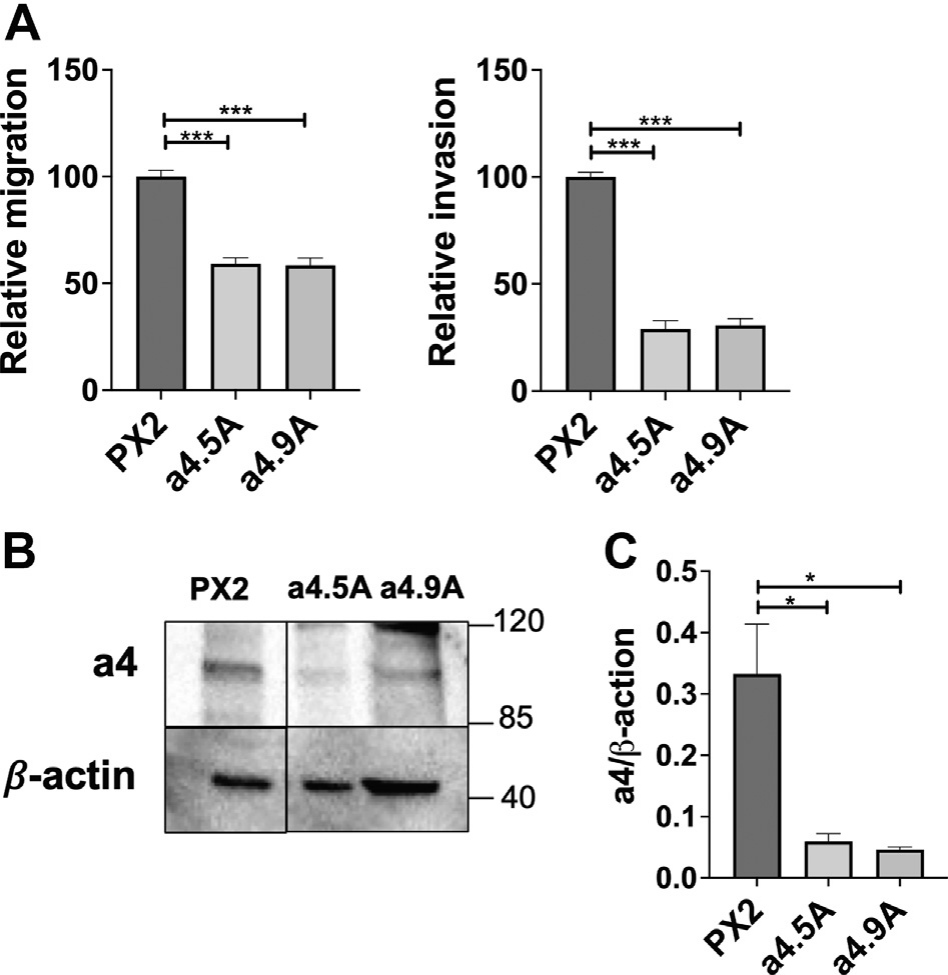
***In vitro* characterization of a4 KO cell lines (a 4.5 A and a 4.9 A).***A*, *in vitro* transwell migration and invasion assays of CRISPR negative control (PX2) and both a 4.5 A and a 4.9 A knockout cell lines were conducted as described in Experimental Procedures. Cells were allowed to migrate through uncoated membranes or to invade through Matrigel-coated membranes toward a chemoattractant. After incubation (18 h for migration and 20 h for invasion), cells were stained with calcein AM, and the number of cells on the *trans* side of the insert were counted in triplicate and averaged. Averages were normalized to the average number of control cells (PX2) counted. n = 2 independent trials; error bars represent SEM (∗∗∗ *p* < 0.0005 calculated using an unpaired two-tailed *t* test with Welch’s correction, comparing each knockout cell line with the control PX2 cell line). *B*, SDS-PAGE and Western blotting analysis of whole-cell lysates collected from CRISPR negative control (PX2) and both a4.5 A and a4.9 A knockout cells lines were performed as described in Experimental Procedures. Antibody against a4 was used to detect expression of the a4-subunit in PX2, a 4.5 A, and a 4.9 A cells lines. β-Actin was used as a protein loading control. Representative image is shown. n = 2. *C*, quantification of Western blots. The intensity of the a4 band was normalized to the amount of protein in each cell lysate using the β-actin band. Each lysate was analyzed in triplicate. n = 2 independent trials. (∗ *p* < 0.05 calculated using an unpaired two-tailed *t* test with Welch’s correction, comparing each knockout cell line with the control PX2 cell line error bars represent SEM).

To further compare the two a4 knockout clones, *in vitro* invasion and migration assays were performed. As can be seen in Fig. 4*A*, the two clones displayed *in vitro* migration and invasion that was reduced by a similar degree relative to control cells, suggesting that the difference observed *in vivo* was due to some property not replicated by the *in vitro* assays. Western blot analysis of the two clones using an a4-specific antibody (Fig. 4, *B* and *C*) also demonstrated that the two a4 knockout clones showed a similar reduction in a4 expression relative to control cells, indicating that a4 expression level alone was not sufficient to explain the difference in tumor growth of these two clones. Additional work will be required to determine the molecular basis for this difference. Nevertheless, both a4 knockout clones showed a dramatically reduced rate of tumor growth relative to control cells.

## Discussion

The primary aim of the present study was to test the hypothesis that V-ATPase a subunit isoforms function in *in vivo* breast cancer metastasis. To address this question, we employed the 4T1-12B murine breast cancer model, which is known to metastasize in immunocompetent mice when implanted orthotopically (32). Here, we describe the first reported *in vivo* use of cancer cells in which individual V-ATPase subunits were selectively disrupted by CRISPR-mediated genome editing.

While parental 4T1-12B cells stably express firefly luciferase (32), it was important to confirm that our various CRISPR-modified clones retained functional luciferase expression. None of the clones tested displayed significant differences in *in vitro* luminescence, meaning the relationship between cell number and luminescence intensity is comparable between clones. We are therefore confident that it is appropriate to directly compare the bioluminescent signals between these clones *in vivo*.

We previously found that in 4T1-12B cells, only CRISPR-mediated disruption of a4, but not other isoforms, significantly reduces V-ATPase localization to the leading edge and also inhibits *in vitro* migration and invasion (21). Importantly, none of the CRISPR-edited cell lines tested displayed significant changes in *in vitro* viability or proliferation (21). Thus, we expected that the a4^−/−^ cells would have a reduced capacity to metastasize in mice but may not have substantial differences in primary tumor growth. It was therefore somewhat surprising that tumor growth was highly impaired in the mice bearing a4^−/−^ allografts. Thus, both CRISPR/Cas9-derived a4^−/−^ clones showed reduced tumor growth compared to negative control cells. This result was not influenced by cell viability, as cell suspensions were confirmed to be ≥95% viable prior to implantation. In addition, palpable tumors were observed within 7 to 10 days of implantation in all but one of the a4^−/−^ mice by IVIS imaging. Comparison of the *in vitro* invasion and migration of the two clones as well as a4 expression levels by Western blot indicated that the observed difference in tumor growth could not be explained on the basis of these *in vitro* properties. Although the basis of the differences between the two a4^−/−^ clones is unclear, differences in phenotype of independent CRISPR clones are not uncommon and are often attributed to either genetic or epigenetic variation between clones (35). It is also possible that one or more clones are subject to off target CRISPR effects (36). Further work will be required to identify the clonal differences responsible for the observed phenotypic differences.

While knockout of a4 caused a significant reduction in tumor growth, knockout of a2 was found to increase tumor growth relative to the control group. a2 is a predominantly intracellular isoform, being found in Golgi, early endosomes, and certain types of secretory vesicles (26, 37, 38). We previously observed that disruption of certain isoforms leads to compensatory expression changes in other isoforms. For example, knockdown of a4 led to increased expression of a3 in MCF10CA1a cells (19). However, knockout of a2 did not affect the expression of any of the other a subunit isoforms in 4T1-12B cells (21). Interestingly, several reports from the Beaman group have found that disruption of a2 in breast cancer cells leads to activation of numerous tumorigenic signaling pathways. While V-ATPase activity is known to promote Notch signaling in *Drosophila* and mammalian cells (39, 40), siRNA-mediated knockdown of a2 increased Notch signaling in invasive MDA-MB-231 and MDA-MB-468 breast cancer cells (41). In the absence of ligand activation, Notch receptors are internalized into sorting endosomes and are then trafficked *via* multivesicular bodies to lysosomes for degradation (42). It has been proposed that V-ATPase inhibition interrupts Notch trafficking into multivesicular bodies and enables cleavage of the Notch intracellular domain, leading to ligand-independent pathway activation (43).

The V-ATPase also promotes Wnt signaling by facilitating internalization of Frizzled receptors into signaling endosomes (44). In MDA-MB-231 and MDA-MB-468 cells, however, knockdown of a2 increased the expression of Wnt target genes. V-ATPase inhibition in some contexts has been suggested to enhance Wnt signaling by preventing autophagic degradation of the Wnt regulatory protein dishevelled (45). Finally, knockdown of a2 in immortalized mammary epithelial cells was found to increase the expression of genes in the TGFβ pathway, which is important for normal mammary gland development and is often dysregulated in breast cancer (46, 47). Unlike Notch and Wnt signaling, it is less clear how V-ATPase inhibition would affect the TGFβ pathway. While TGFβ signaling does not require endocytosis, it appears to be enhanced by internalization of TGFβ receptors into signaling endosomes (48). V-ATPase inhibition may block further trafficking and degradation of internalized receptors, thus enhancing or prolonging TGFβ pathway activation. Additionally, TGFβ is a positive regulator of autophagy (49, 50). Upon inhibition of autophagic flux in HK-2 cells using the V-ATPase inhibitor bafilomycin A, TGFβ levels increase because of decreased autolysosomal TGFβ degradation (51). This likely serves as a feedback mechanism to increase levels of autophagy. Knockdown of a2 could likewise be decreasing autophagic flux which in turn may increase TGFβ signaling for the same purpose.

As our primary aim was to study the role of a isoforms in metastasis, we employed a luciferase expressing model to continuously monitor the spread of tumor cells throughout the body. Unfortunately, the strong luminescence signal from the primary tumor prevented detection of signals from metastases *in vivo*. Metastases were evaluated by biophotonic imaging of isolated organs following sacrifice of all mice in the second trial. Compared to the control group, in which 15 out of 15 mice showed lung metastases, only one out of 19 of the a4 knockout mice showed metastases to lung (this included mice from both a4 knockout clones). Interestingly, knockout of a1 also partially reduced the number of lung metastases (3 out of 8). Although no difference in the number of mice showing bone metastases was observed for the a4 knockout mice relative to the control group (Table 1), there was a significant reduction in the size and measured luminescence of the bone metastases in the a4 knockout mice (Fig. 4). This could be explained by either a delay in the ability of tumor cells to metastasize to bone (metastases were evaluated at the endpoint, which could disguise temporal differences in metastasis) or a reduced rate of growth of bone metastases. Consistent with the latter possibility, the a4 knockout clone showing smaller bone metastases was also the clone showing lower growth of the primary tumor. Further experiments will be required to distinguish between these possibilities. Although a reduced number of metastases were observed at other sites for the control cells, including spleen, liver, kidney, and brain, no metastases were observed at any of these sites for the a4 knockout cells. It should be noted that the reduced tumor growth of the a4 knockout clones may be a significant contributor to the observed reductions in metastases for these clones.

Concerning why knockout of a4 does not prevent metastasis to bone, it is known that metastasis of breast tumor cells to bone is significantly dependent upon osteoclasts (52). It is possible that a4 knockout cells are as efficient as normal cancer cells at recruiting osteoclasts for metastasis to bone and therefore show the same number of metastases. By contrast, for other tissues where metastasis is directly dependent upon the ability tumor cells themselves to invade, reduced metastasis is observed for the a4 knockout cells.

It is also interesting that a1 knockout cells showed a partial reduction in the number of lung metastases and both a1 and a3 knockout cells showed reduced size of bone metastases. We previously observed a partial reduction in a4 expression in cells in which a1 had been knocked out, although no difference in *in vitro* invasion or migration was observed (21). It is possible that this partial reduction in a4 expression nevertheless resulted in reduced metastases to lung and reduced ability of metastases to grow in bone *in vivo*, despite showing only a minor effect on growth of the primary tumor. By contrast, a3 knockout cells did not display any reduction in a4 expression (21). Moreover, the a3 disruption was only partial in these cells (21), suggesting an especially important role of a3 in the growth of metastases in bone. Because of the important role a3 has been shown to play in invasion and migration of other breast cancer cell lines (16, 18, 19), it is possible that a3 plays a similar role in the growth of 4T1-12B metastases in bone, despite its apparent lack of function in migration and invasion of these cells *in vitro*. Such a result emphasizes the importance of determining the properties of tumor cells in an *in vivo* context. Interestingly, comparison of human breast tumor samples by quantitative RT-PCR revealed that all 43 samples tested showed upregulation of a3 mRNA relative to control samples (16).

It is highly encouraging that primary tumor growth was markedly impaired in mice bearing a4^−/−^ allografts. As discussed earlier, this was somewhat surprising because the a4^−/−^ cells did not display differences in viability, measured by trypan blue, or proliferation, measured by WST-1 (21). There are obviously many differences between two-dimensional growth on plastic and three-dimensional growth *in vivo*. One major difference is that, *in vivo*, 4T1 tumors often become necrotic upon reaching a macroscopic size. A major cause of tumor necrosis is hypoxia, resulting from insufficient vascularization (53). Under hypoxic conditions, tumor cells generate excess metabolic acid, which must be removed from the cytoplasm (54). Since the potential for proton sequestration within intracellular compartments is relatively limited, it is likely that proton transport across the plasma membrane is required for cancer cell survival in extreme hypoxia. V-ATPases are the major plasma membrane proton transporters in cancer cells, and we have previously shown that selective inhibition of plasma membrane V-ATPases in MDA-MB-231 breast cancer cells significantly decreases cytosolic pH (20). Since disruption of a4, but not other isoforms, significantly reduced plasma membrane V-ATPase localization in 4T1-12B cells, it is likely that a4^−/−^ 4T1-12B cells have a reduced ability to remove cytosolic acid *in vivo* (21). This is further supported by our observation that only knockout of a4 significantly reduces plasma membrane proton flux in 4T1-12B cells, as measured by Seahorse ECAR assay (21) By contrast, cells cultured under standard *in vitro* conditions are never hypoxic, as they are grown in room air (21% O_2_) supplemented with 5% CO_2_ (55). Future studies using hypoxia chambers will thus be needed to determine whether a4 (or plasma membrane V-ATPases in general) is important for tumor cell growth in hypoxic conditions.

## Experimental procedures

### Cell culture

The CRISPR-modified cells used in this study were derived previously (21). Cells were maintained in Dulbecco's modified Eagle's medium (DMEM) (Gibco #11995) supplemented with 10% fetal bovine serum (FBS) (Sigma-Aldrich #12306C) and 1% penicillin/streptomycin (Gibco #15140). Cells were grown on tissue culture–treated polystyrene dishes and routinely passaged by detaching with 0.25% trypsin-EDTA. Cells were maintained in a humidified environment at 37 °C with 5% CO_2_. Cell lines were discarded upon reaching passage 15.

### Cell viability

Cell viability was measured using 0.4% Trypan Blue Solution (Gibco #15250061) according to the manufacturer’s instructions. Briefly, cell suspensions were mixed 1:1 with 0.4% Trypan Blue Solution, and the number of cells taking up the dye was counted using a hemocytometer. The following formula was used to determine cell viability: % viable cells = [(total cells – blue cells) ÷ total cells] × 100.

### *In vitro* luciferase assay

Increasing densities of WT, negative CRISPR control and a isoform knockout clones were seeded in triplicate wells of white, opaque 96-well tissue culture microplates and allowed to attach overnight. The following day, the growth media were replaced with fresh media containing 150 μg/ml d-luciferin monopotassium salt (Promega #E1601), and luminescence was immediately measured using a Tecan SpectraFluor Plus Microplate Reader at 37 °C with 1s integration time. The results from two independent trials were used for statistical analysis.

### *In vivo* metastasis model

Negative CRISPR control and a isoform knockout 4T1-12B cells were seeded at 1 × 10^5^/cm^2^ (approximately 40% confluency) and allowed to attach overnight. The following day, cells were detached by trypsinization and verified to be ≥95% viable by trypan blue exclusion. Cells were then centrifuged at 300×*g* for 5 min, and the cell pellets were resuspended in fresh DMEM at 1 × 10^7^ cells/ml. Hundred microliter of the resulting cell suspension was injected into the intact no. 4 inguinal fat pads of 6-week-old female BALB/c mice using a 26-gauge needle. Primary tumor dimensions were acquired weekly by caliper measurement, and tumor volume was calculated using the modified ellipsoid formula (L × W^2^)/2 (56).

### Bioluminescent imaging

*In vivo* imaging was performed on the first day postimplantation to verify accurate implantation of cells. A fresh luciferin solution was prepared by dissolving XenoLight d-luciferin monopotassium salt (PerkinElmer #122799) in PBS at 10 mg/ml. The luciferin solution was filter-sterilized, and 100 μl was injected into each mouse intraperitoneally. Mice were anesthetized with a 2.5%/97.5% isoflurane/O_2_ mixture using a Caliper Life Sciences XGI-8 Gas Anesthesia System and imaged 10 min postluciferin injection using a PerkinElmer IVIS SpectrumCT *In vivo* Imaging System. At the conclusion of the study (or when humane endpoints were reached), animals were sacrificed, and organs were removed and imaged *ex vivo* following the same luciferin injection protocol. Because the intensity of the signal from the primary tumor prevented detection of metastases in the intact mice, live animal imaging was not performed after the initial imaging described.

### Animal care

Female BALB/c mice aged 6 weeks were purchased from The Jackson Laboratory and housed within the Tufts University animal facility. All animal work was approved by, and carried out in accordance with, the Tufts University Institutional Animal Care and Use Committee. Mice (where indicated) were euthanized when they reached the predetermined endpoint criterion of tumor volume greater than 1500 mm^3^. Two mice in the a2^−/−^ were euthanized because of decreased body condition, increased respiratory rate, and hunched posture. No mice were found to have body weight loss ≥ 15% from baseline. One mouse in the a2^−/−^ group was found dead at week 5, and one mouse in the a4^−/−^ group was found dead at week 6.

### Transwell migration assay

*In vitro* transwell migration assays were performed as described previously (16). Briefly, the assay was performed using Fluoroblok inserts (Corning Falcon #351152) with an 8-μm pore size were placed into a 24 well plate containing 500 μl of DMEM (Gibco #11995) with 10% FBS (Sigma-Aldrich #12306C) in each well. Cells were trypsinized and brought to a concentration of 1.5 × 10^5^ cells/ml in filter-sterilized DMEM containing 0.1% BSA (Sigma-Aldrich #A7030–50G). Five hundred microliters of the cell suspension was added to the *cis* side of the membranes of the Fluorblok inserts. Each cell type or treatment was done in triplicate. Cells were incubated at 37 °C in 5% CO_2_. After 18 h incubation, the membrane inserts were placed into wells containing 4 μg/ml calcein AM (Sigma-Aldrich #17783) diluted in PBS (Gibco #10010023) and incubated for 5 min at 37 °C in 5% CO_2_. Cells that had migrated to the *trans* side of the membrane were imaged using a Nikon Eclipse TE2000-S fluorescence microscope. An average of eight images were taken per well, and the number of migrating cells was averaged over three wells.

### Transwell invasion assay

*In vitro* transwell invasion assays were performed as described previously (19). Briefly, the assay was performed using Fluoroblok inserts (Corning Falcon #351152) with an 8-μm pore size that were coated with Matrigel (Corning). Sixty microliters of Matrigel diluted to a final concentration of 0.2 μg/μl in PBS was added to each membrane. Membranes were allowed to dry overnight under vacuum at room temperature. Matrigel-coated membranes were rehydrated with 60 μl DMEM for at least 2 h, and 500 μl of DMEM (Gibco #11995) with 10% FBS (Sigma-Aldrich #12306C) were added to wells in a 24-well plate to act as a chemoattractant, and Matrigel coated inserts were placed in each well. Cells were trypsinized and brought to a concentration of 1.5 × 10^5^ cells/ml in filter-sterilized DMEM containing 0.1% BSA (Sigma-Aldrich #A7030–50G). Five hundred microliters of the cell suspension was added to the *cis* side of the Matrigel-coated membranes of the Fluorblok inserts. Each cell type was done in triplicate. Cells were incubated at 37 °C in 5% CO_2 incubator_. After 20 h incubation, the membrane inserts were placed into wells containing 4 μg/ml calcein AM (Sigma-Aldrich #17783) diluted in PBS (Gibco #10010023) and incubated for 5 min at 37 °C in 5% CO_2_. Cells that had invaded to the *trans* side of the Matrigel-coated membrane were imaged using a Nikon Eclipse TE2000-S fluorescence microscope. An average of eight images were taken per well, and the number of invading cells was averaged over three wells.

### Whole-cell lysis and Western blotting

When cells reached about 80 % confluence, they were placed on ice and washed two times with ice-cold PBS. Cells were scraped into 450 μl of ice-cold lysis buffer supplemented with protease inhibitors (150 mM NaCl, 1% Triton X-100, 50 mM Tris-HCl (pH 7.5), 1 mM phenylmethylsulfonyl fluoride (Sigma #P7626), 2 μg/ml aprotinin (Roche Applied Science #10981532001), 5 μg/ml leupeptin (Thermo Fisher Scientific #78435), 1 μg/ml pepstatin (Roche Applied Science #11524488001), 1 mM NaF, and 1 mM glycerophosphate). Cell lysates were subjected to continuous agitation for 30 min to lyse the cells followed by centrifugation at 500×*g* for 10 min to remove cellular debris.

Following whole-cell lysis, protein concentrations were determined using the Lowry method (57). SDS-containing sample buffer was used to dilute the lysate. Proteins were separated by SDS-PAGE on 4 to 15% precast gels (Bio-Rad # 456–1084), transferred onto a nitrocellulose membrane, and blocked with 1.5% milk in TBS-T. After blocking, membranes were incubated with primary antibodies overnight at 4 °C or at room temperature for at least 1 h. After incubation with primary antibodies, the membrane was washed with TBS-T and incubated with the appropriate secondary antibodies in milk for 1 h. Protein detection was performed using chemiluminescent substrate (Thermo Fisher Scientific), imaged, and quantified using Chemidoc XRS+ with Image Lab 6.0.1 software (Bio-Rad). The intensity of the a4 band was normalized to the amount of protein in each lane using the β-actin band. Primary antibodies used were mammalian anti-V_0_a4 (Abcam # ab97440; 1:1000) and anti-β-actin- HRP (Cell Signaling # 5125; 1:1000). Secondary antibody used was anti-rabbit-HRP (Cell Signaling # 7074; 1:10,000).

### Statistics

The *p* values were calculated using an unpaired two-tailed *t* test with Welch’s correction, comparing each knockout cell line with the control PX2 cell line.

## Data availability

All data are contained within the manuscript.

## Supporting information

This article contains supporting information (57).

## Conflict of interest

The authors declare that they have no conflicts of interest with the contents of this article.

## References

[1] Bray F., Ferlay J., Soerjomataram I., Siegel R.L., Torre L.A., Jemal A. (2018). Global cancer statistics 2018: GLOBOCAN estimates of incidence and mortality worldwide for 36 cancers in 185 countries. CA. Cancer J. Clin..

[2] Harbeck N., Penault-Llorca F., Cortes J., Gnant M., Houssami N., Poortmans P. (2019). Breast cancer. Nat. Rev. Dis. Primer..

[3] Valastyan S., Weinberg R.A. (2011). Tumor metastasis: Molecular insights and evolving paradigms. Cell.

[4] Massagué J., Obenauf A.C. (2016). Metastatic colonization by circulating tumour cells. Nature.

[5] Welch D.R., Hurst D.R. (2019). Defining the hallmarks of metastasis. Cancer Res..

[6] Stransky L., Cotter K., Forgac M. (2016). The function of V-ATPases in cancer. Physiol. Rev..

[7] Collins M.P., Forgac M. (2018). Regulation of V-ATPase assembly in nutrient sensing and function of V-ATPases in breast cancer metastasis. Front. Physiol..

[8] Forgac M. (2007). Vacuolar ATPases: Rotary proton pumps in physiology and pathophysiology. Nat. Rev. Mol. Cell Biol..

[9] Kane P.M. (2007). The long physiological reach of the yeast vacuolar H^+^-ATPase. J. Bioenerg. Biomembr..

[10] Breton S., Brown D. (2013). Regulation of luminal acidification by the V-ATPase. Physiol. Bethesda Md..

[11] Sun-Wada G.-H., Wada Y. (2013). Vacuolar-type proton pump ATPases: Acidification and pathological relationships. Histol. Histopathol..

[12] Holliday L.S. (2014). Vacuolar H^+^-ATPase: An essential multitasking enzyme in physiology and pathophysiology. New J. Sci..

[13] Cotter K., Stransky L., McGuire C., Forgac M. (2015). Recent insights into the structure, regulation, and function of the V-ATPases. Trends Biochem. Sci..

[14] Oot R.A., Couoh-Cardel S., Sharma S., Stam N.J., Wilkens S. (2017). Breaking up and making up: The secret life of the vacuolar H^+^-ATPase. Protein Sci. Publ. Protein Soc..

[15] Vasanthakumar T., Rubinstein J.L. (2020). Structure and roles of V-type ATPases. Trends Biochem. Sci..

[16] Cotter K., Liberman R., Sun-Wada G., Wada Y., Sgroi D., Naber S. (2016). The a3 isoform of subunit a of the vacuolar ATPase localizes to the plasma membrane of invasive breast tumor cells and is overexpressed in human breast cancer. Oncotarget.

[17] Sennoune S.R., Bakunts K., Martínez G.M., Chua-Tuan J.L., Kebir Y., Attaya M.N. (2004). Vacuolar H^+^-ATPase in human breast cancer cells with distinct metastatic potential: Distribution and functional activity. Am. J. Physiol. Cell Physiol..

[18] Hinton A., Sennoune S.R., Bond S., Fang M., Reuveni M., Sahagian G.G. (2009). Function of a subunit isoforms of the V-ATPase in pH homeostasis and *in vitro* invasion of MDA-MB231 human breast cancer cells. J. Biol. Chem..

[19] Capecci J., Forgac M. (2013). The function of vacuolar ATPase (V-ATPase) a subunit isoforms in invasiveness of MCF10a and MCF10CA1a human breast cancer cells. J. Biol. Chem..

[20] Cotter K., Capecci J., Sennoune S., Huss M., Maier M., Martinez-Zaguilan R. (2015). Activity of plasma membrane V-ATPases is critical for the invasion of MDA-MB231 breast cancer cells. J. Biol. Chem..

[21] McGuire C.M., Collins M.P., Sun-Wada G., Wada Y., Forgac M. (2019). Isoform-specific gene disruptions reveal a role for the V-ATPase subunit a4 isoform in the invasiveness of 4T1-12B breast cancer cells. J. Biol. Chem..

[22] Wiedmann R.M., von Schwarzenberg K., Palamidessi A., Schreiner L., Kubisch R., Liebl J. (2012). The V-ATPase-inhibitor archazolid abrogates tumor metastasis via inhibition of endocytic activation of the Rho-GTPase Rac1. Cancer Res..

[23] Schempp C.M., von Schwarzenberg K., Schreiner L., Kubisch R., Müller R., Wagner E. (2014). V-ATPase inhibition regulates anoikis resistance and metastasis of cancer cells. Mol. Cancer Ther..

[24] Toyomura T., Oka T., Yamaguchi C., Wada Y., Futai M. (2000). Three subunit a isoforms of mouse vacuolar H^+^-ATPase. Preferential expression of the a3 isoform during osteoclast differentiation. J. Biol. Chem..

[25] Smith A.N., Skaug J., Choate K.A., Nayir A., Bakkaloglu A., Ozen S. (2000). Mutations in ATP6N1B, encoding a new kidney vacuolar proton pump 116-kD subunit, cause recessive distal renal tubular acidosis with preserved hearing. Nat. Genet..

[26] Pietrement C., Sun-Wada G.-H., Silva N.D., McKee M., Marshansky V., Brown D. (2006). Distinct expression patterns of different subunit isoforms of the V-ATPase in the rat epididymis. Biol. Reprod..

[27] Dexter D.L., Kowalski H.M., Blazar B.A., Fligiel Z., Vogel R., Heppner G.H. (1978). Heterogeneity of tumor cells from a single mouse mammary tumor. Cancer Res..

[28] Heppner G.H., Dexter D.L., DeNucci T., Miller F.R., Calabresi P. (1978). Heterogeneity in drug sensitivity among tumor cell subpopulations of a single mammary tumor. Cancer Res..

[29] Miller F.R., Miller B.E., Heppner G.H. (1983). Characterization of metastatic heterogeneity among subpopulations of a single mouse mammary tumor: Heterogeneity in phenotypic stability. Invasion Metastasis.

[30] Gómez-Cuadrado L., Tracey N., Ma R., Qian B., Brunton V.G. (2017). Mouse models of metastasis: Progress and prospects. Dis. Model. Mech..

[31] Park M.K., Lee C.H., Lee H. (2018). Mouse models of breast cancer in preclinical research. Lab. Anim. Res..

[32] Tao K., Fang M., Alroy J., Sahagian G.G. (2008). Imagable 4T1 model for the study of late stage breast cancer. BMC Cancer.

[33] DuPre’ S.A., Hunter K.W. (2007). Murine mammary carcinoma 4T1 induces a leukemoid reaction with splenomegaly: Association with tumor-derived growth factors. Exp. Mol. Pathol..

[34] Arroyo-Crespo J.J., Armiñán A., Charbonnier D., Deladriere C., Palomino-Schätzlein M., Lamas-Domingo R. (2019). Characterization of triple-negative breast cancer preclinical models provides functional evidence of metastatic progression. Int. J. Cancer.

[35] Khan G.N., Kim E.J., Shin T.S., Lee S.H. (2017). Heterogeneous cell types in single-cell-derived clones of MCF7 and MDA-MB-231 cells. Anticancer Res..

[36] Zhang X.-H., Tee L.Y., Wang X.-G., Huang Q.-S., Yang S.-H. (2015). Off-target effects in CRISPR/Cas9-mediated genome engineering. Mol. Ther. - Nucleic Acids.

[37] Toyomura T., Murata Y., Yamamoto A., Oka T., Sun-Wada G.-H., Wada Y. (2003). From lysosomes to the plasma membrane: Localization of vacuolar-type H^+^-ATPase with the a3 isoform during osteoclast differentiation. J. Biol. Chem..

[38] Hurtado-Lorenzo A., Skinner M., El Annan J., Futai M., Sun-Wada G.-H., Bourgoin S. (2006). V-ATPase interacts with ARNO and Arf6 in early endosomes and regulates the protein degradative pathway. Nat. Cell Biol..

[39] Yan Y., Denef N., Schüpbach T. (2009). The vacuolar proton pump, V-ATPase, is required for Notch signaling and endosomal trafficking in *Drosophila*. Dev. Cell..

[40] Sethi N., Yan Y., Quek D., Schupbach T., Kang Y. (2010). Rabconnectin-3 is a functional regulator of mammalian Notch signaling. J. Biol. Chem..

[41] Pamarthy S., Jaiswal M.K., Kulshreshtha A., Katara G.K., Gilman-Sachs A., Beaman K.D. (2015). The vacuolar ATPase a2-subunit regulates Notch signaling in triple-negative breast cancer cells. Oncotarget.

[42] Le Borgne R. (2005). The roles of receptor and ligand endocytosis in regulating Notch signaling. Development.

[43] Bray S.J. (2016). Notch signalling in context. Nat. Rev. Mol. Cell Biol..

[44] Cruciat C.-M., Ohkawara B., Acebron S.P., Karaulanov E., Reinhard C., Ingelfinger D. (2010). Requirement of prorenin receptor and vacuolar H^+^-ATPase-mediated acidification for Wnt signaling. Science.

[45] Gao C., Cao W., Bao L., Zuo W., Xie G., Cai T. (2010). Autophagy negatively regulates Wnt signalling by promoting Dishevelled degradation. Nat. Cell Biol..

[46] Pamarthy S., Mao L., Katara G.K., Fleetwood S., Kulshreshta A., Gilman-Sachs A. (2016). The V-ATPase a2 isoform controls mammary gland development through Notch and TGF-β signaling. Cell Death Dis.

[47] Massagué J. (2008). TGFβ in cancer. Cell.

[48] Chen Y.-G. (2009). Endocytic regulation of TGF-β signaling. Cell Res.

[49] Zhang Y., Alexander P.B., Wang X.-F. (2017). TGF-β family signaling in the control of cell proliferation and survival. Cold Spring Harb. Perspect. Biol..

[50] Alizadeh J., Glogowska A., Thliveris J., Kalantari F., Shojaei S., Hombach-Klonisch S. (2018). Autophagy modulates transforming growth factor beta 1 induced epithelial to mesenchymal transition in non-small cell lung cancer cells. Biochim. Biophys. Acta BBA - Mol. Cell Res..

[51] Ding Y., Kim S. ll, Lee S.-Y., Koo J.K., Wang Z., Choi M.E. (2014). Autophagy regulates TGF- *β* expression and suppresses kidney fibrosis induced by unilateral ureteral obstruction. J. Am. Soc. Nephrol..

[52] Croucher P.I., McDonald M.M., Martin T.J. (2016). Bone metastasis: The importance of the neighbourhood. Nat. Rev. Cancer.

[53] Proskuryakov S.Y., Gabai V.L. (2010). Mechanisms of tumor cell necrosis. Curr. Pharm. Des..

[54] Webb B.A., Chimenti M., Jacobson M.P., Barber D.L. (2011). Dysregulated pH: A perfect storm for cancer progression. Nat. Rev. Cancer.

[55] Al-Ani A., Toms D., Kondro D., Thundathil J., Yu Y., Ungrin M. (2018). Oxygenation in cell culture: Critical parameters for reproducibility are routinely not reported. PLoS One.

[56] Tomayko M.M., Reynolds C.P. (1989). Determination of subcutaneous tumor size in athymic (nude) mice. Cancer Chemother. Pharmacol..

[57] Lowry O.H., Rosebrough N.J., Farr A.L., Randall R.J. (1951). Protein measurement with the Folin phenol reagent. J. Biol. Chem..

