# Supplementary material for: Isoform a4 of the vacuolar ATPase a subunit promotes 4T1-12B breast cancer cell–dependent tumor growth and metastasis in vivo
Source: J Biol Chem. 2022 Aug 19;298(10):102395. doi: 10.1016/j.jbc.2022.102395 (PMC9508560; doi:10.1016/j.jbc.2022.102395)
Supplement: Supporting Information [file mmc1.pdf]

Isoform a4 of the vacuolar ATPase a subunit promotes 4T1-12B breast cancer cell-dependent tumor growth and metastasis *in vivo*

Kevin Su [#,4,5], Michael P. Collins [#,2,6], Christina M. McGuire [3,7], Mohammed A. Alshagawi [4], Mariam K. Alamoudi [1,8] Zhen Li [4] and Michael Forgac [1,2,3,4, *]

Experimental procedures:

In vivo metastasis model

Negative CRISPR control and a isoform knockout 4T1-12B cells were seeded at $1\times10^5/cm^2$ (approximately 40% confluency) and allowed to attach overnight. The following day, cells were detached by trypsinization and verified to be ≥95% viable by trypan blue exclusion. Cells were then centrifuged at 300×*g* for 5 minutes, and the cell pellets were resuspended in fresh DMEM at 1×10^7 cells/ml. 100µl of the resulting cell suspension were injected into the intact no. 4 inguinal fat pads of 6-week old female BALB/c mice using a 26-gauge needle. Primary tumor dimensions were acquired weekly by caliper measurement, and tumor volume was calculated using the modified ellipsoid formula $(L \times W^2)/2$ (57).

Bioluminescent imaging

In vivo imaging was performed on the first day post-implantation to verify accurate implantation of cells. A fresh luciferin solution was prepared by dissolving XenoLight D-luciferin monopotassium salt (Perkin Elmer #122799) in PBS at 10mg/ml. The luciferin solution was filter-sterilized, and 100µl was injected into each mouse intraperitoneally. Mice were anesthetized with a 2.5%/97.5% isoflurane/O_2 mixture using a Caliper Life Sciences XGI-8 Gas Anesthesia System, and imaged 10 minutes post-luciferin injection using a Perkin Elmer IVIS SpectrumCT In Vivo Imaging System. At the conclusion of the study (or when humane endpoints were reached), animals were sacrificed, and organs were removed and imaged *ex vivo* following the same luciferin injection protocol. Because the intensity of the signal from the primary tumor prevented detection of metastases in the intact mice, live animal imaging was not performed after the initial imaging described.

Animal care

Female BALB/c mice aged 6 weeks were purchased from The Jackson Laboratory (Bar Harbor, ME), and housed within the Tufts University animal facility. All animal work was approved by, and carried out in accordance with, the Tufts University Institutional Animal Care and Use Committee. Mice (where indicated) were euthanized when they reached the predetermined endpoint criterion of tumor volume greater than $1500mm^3$. Two mice in the $a2^{-/-}$ were euthanized because of decreased body condition, increased respiratory rate and hunched posture. No mice were found to have body weight loss ≥ 15% from baseline. One mouse in the $a2^{-/-}$ group was found dead at week 5, and one mouse in the $a4^{-/-}$ group was found dead at week 6.

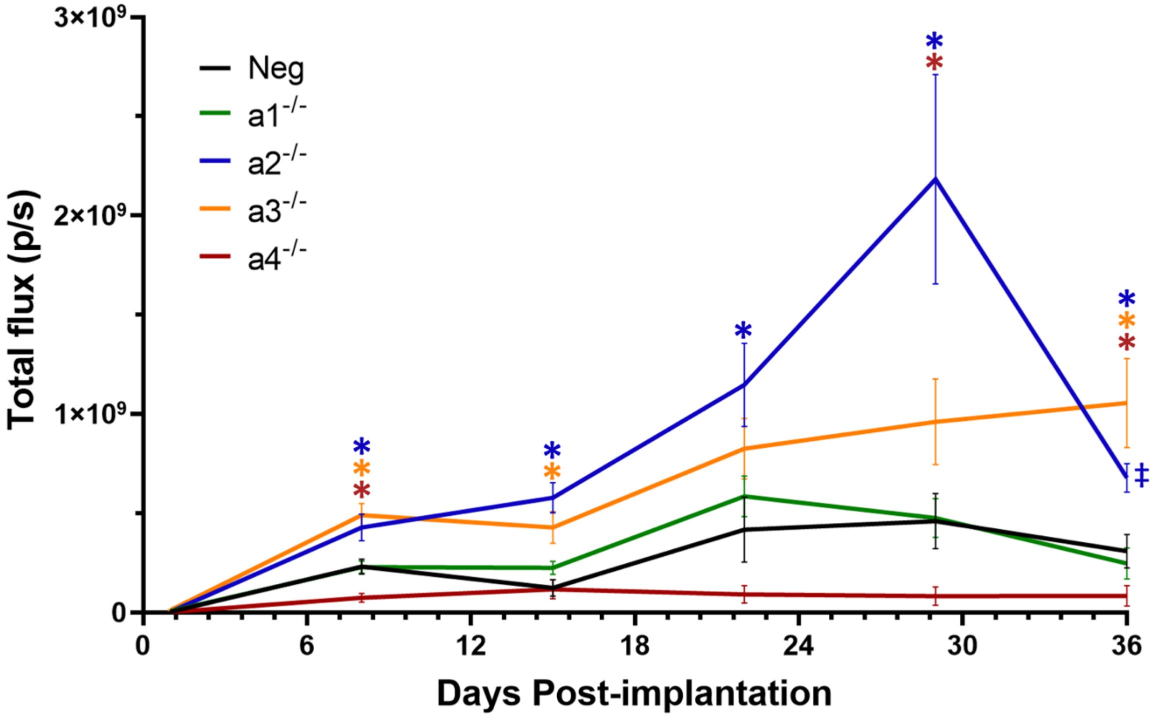

Figure S.1. ***Primary Tumor bioluminescence in Blab/c mice bearing negative control and CRISPR mediated a subunit isoform knockout 4T1-12B allografts.*** Whole animal bioluminescence was performed at the indicated times following tumor cell implantation for mice in Experiment 1 (Fig.2) as described in Experimental procedures. Luminescence is expressed as total flux (p/s) for the region of interest (ROI) set to detect the primary tumor. Values represent the mean. n = 10, unless otherwise noted. * $p < 0.05$ for indicated knockout relative to PX2 control; at day 22, a4 KO has a p value of 0.069 relative to control; ‡ n = 6. Error bars represent SEM.
